# Supplementary material for: Pathogenic PDE12 variants impair mitochondrial RNA processing causing neonatal mitochondrial disease
Source: EMBO Mol Med. 2024 Nov 20;17(1):193–210. doi: 10.1038/s44321-024-00172-5 (PMC11729904; doi:10.1038/s44321-024-00172-5)
Supplement: Supplementary file 1 — Appendix [file 44321_2024_172_MOESM1_ESM.pdf]

Appendix Supplementary Information

Pathogenic PDE12 variants impair mitochondrial RNA processing causing neonatal-onset mitochondrial disease: supplementary information

Table of contents: Appendix

Appendix Family 1 .... page 1

Appendix Family 2 .... page 2

Appendix Family 3 .... page 4

Family 1:

Patient 1 (female) was born as the first child of consanguineous (1st cousin) parents and died at 3 months of life. Patient 2, a male sibling, was born at term by emergency lower segment Caesarean section (LSCS) for suspected placental abruption. Antenatally there was massive polyhydramnios, requiring two amnioreductions during the pregnancy, and one amnioreduction (>2 liters) immediately prior to birth. Patient 2 appeared well soon after birth, but deteriorated at 3 minutes of life, with poor respiratory effort requiring positive pressure ventilation. He was brought to the neonatal intensive care unit and was noted to have poor respiratory effort, poor muscle tone with oxygen saturations of 80% and respiratory acidosis. He was subsequently intubated and ventilated. He was extubated to CPAP on day 3 of life but continued to require intermittent pressure support via CPAP and then high-flow O2, until day 56 of life.

Patient 2 was noted to have persistent lactic acidosis with a maximum lactate of 7 mmol/L (normal, <2.2 mmol/L). Plasma amino acids on DOL 1 showed a relative increase in Glycine with most other amino acids below or at the lower end of the reference ranges and urine organic acids on DOL 2 showed non-specific elevations of hexanoate lactones in the absence of detectable 4-

hydroxybutyrate. Repeat urine organics on DOL 13 showed a mild increase in the excretion of Krebs cycle intermediates (citrate, 2-oxoglutarate, fumarate, malate, succinate). A mild increase in 3-hydroxyglutarate with no associated increase in glutarate was also detected. Acylcarnitines were normal. A muscle biopsy revealed a mosaic pattern of COX deficiency with approximately 60% COX-deficient fibers, while assessment of respiratory chain enzyme activities revealed combined CI and IV defects, confirming diagnosis of mitochondrial disease.

An MRI of the brain, performed at day 21 of life, revealed small areas of prominent perivascular spaces in the inferior portion of the basal ganglia, a nonspecific finding. The cisterna magna and frontotemporal cerebrospinal fluid spaces were slightly prominent. Patient 2 slowly improved with support of feeding therapy and was discharged from the Hospital on day 68 of life.

Following discharge, he was noted to have mild developmental delays, performing at 2-year-level at age 3.5 years. Currently he is 7 years old; he has struggled to gain weight, despite being given high calorie foods. His weight is at the 9th centile while height has recently dropped from the 25th to the 9th centiles. Head circumference is at the 50th centile. His development continues to be ~ 18-24 months behind his chronological age. He fatigues easily on walking but has otherwise normal physical exam. Lactate levels have normalized after the neonatal period and liver and kidney function tests are normal.

Family 2:

A male infant (Patient 3) was born at 39 weeks via elective caesarean section secondary to breech presentation. He was the first baby to a non-consanguineous couple with otherwise negative family history. Mother reported decreased fetal movements towards the end of the pregnancy and a prenatal ultrasound was concerning for brain abnormalities. The baby was limp and apneic at birth, requiring resuscitation with positive pressure ventilation. Birth weight was 2.3kg (small for

gestational age). Apgar scores were 1, 6 and 7 at 1, 5 and 10 minutes respectively. Initial blood gas revealed mixed respiratory & metabolic acidosis with pH of 7.1, pCO2 of 58, and BE of -11 although perfusion and blood pressure were adequate. With respiratory support, pCO2 normalized, but metabolic acidosis worsened with pH decreasing to 6.9. Patient soon became apnoeic and was intubated at the birth hospital and transferred to tertiary centre for higher level care.

On arrival, the patient had no spontaneous movements or respiratory effort. Repeat blood gas showed severe acidosis with pH of 7.01, pCO2 of 38, BE of -19, lactic acid of 16.8 mmol /L (<2.1), normal ammonia at 43 μmol/L (<75 μmol/L) and mildly elevated β-hydroxybutyric acid at 6.4 mg/dl (< 2.8 mg/dl). Repeat labs showed increased lactic at 20 mmol/L and pyruvate at 0.33 mmol/L (0.03-0.08 mmol/L) with elevated L/P ratio of 60.6 (<20). Plasma aminoacids were significant for elevated alanine at 1,966 μmol/L (131- 710 μmol/L), and mild elevations in glutamine, proline and lysine with normal citrulline. Urine organic acids showed very large increase in lactate, with moderate increase in pyruvate and 4-hydroxyphenyllactic, and mild increase in 3-hydroxybutyrate and 2-hydroxybutyrate. Total and free carnitine and acylcarnitine profile, were normal. Sepsis work-up was negative, brain natriuretic peptide (BNP) and troponin were mildly elevated, but echocardiogram was normal.

An MRI of the brain was striking for lissencephaly, severe dysgenesis of the corpus callosum, and extensive periventricular and subcortical cysts (**Figure 1**). A region of restricted diffusion in the left basal ganglia was also visualized (not shown). Severe lactic acidosis (pH 6.8-7.1, Lactic acid 11.99-20.0) persisted despite aggressive treatment with IV sodium acetate, bicarbonate, thiamine (100 mg/kg/day IV) and carnitine (100 mg/kg/day IV). Patient developed severe hyperglycemia (blood glucose over 400) which did not respond to lowering glucose infusion rate or IV insulin drip. On second day of life, parents elected to discontinue support and patient died shortly thereafter.

As skin biopsy was obtained for fibroblast culture. Pyruvate dehydrogenase complex and electron transfer chain activities in fibroblasts were normal.

Family 3:

Parents are first cousins and have no significant medical or family history. Their first pregnancy resulted in a healthy boy. The 2nd pregnancy (Foetus 4) was complicated by abnormal ultrasound during the first trimester revealing increased nuchal translucency at 8mm, megacystis, and subcutaneous edema. At 19 gestational week (gw) and 5d ays, severe intrauterine growth retardation, hydrops and cystic hygroma were observed. Three days later, a fetal ultrasound additionally revealed hypertrophy of the right ventricle, decreased fetal movements, lung hypoplasia, talipes equinovarus and suspected vermis rotation. The pregnancy ended spontaneously by miscarriage at 22gw. Fetal examination revealed a male fetus with intrauterine growth retardation, cystic hygroma, and facial dysmorphisms with hypertelorism, high and large forehead and low set ears (**Fig EV1A-C**). He presented fetal hydrops, and bilateral knee and elbow ankyloses (**Fig EV1D,E**). Autopsy showed mild tricuspid stenosis, pulmonary hypoplasia and distended bladder (**Fig EV1F**). Neuropathological examination could not confirm a vermis anomaly. Microarray chromosomal analysis was normal.

During the 3rd pregnancy (Foetus 5) a fetal ultrasound performed at 13gw and two days revealed increased nuchal translucency (14mm), permanent flexion contractures of four limbs and absence of fetal movements. Termination of pregnancy was performed 13 gw and six days for recurrence of fetal akinesia. Microarray chromosomal analysis was normal. Fetal examination showed severe intrauterine growth retardation (biometry at the 5th centile), confirmed contractures of four limbs with arthrogryposis, multiple pterygia (colli, elbows, knees) and generalized muscular atrophy (**Fig**

EV1G-H). Histological analysis showed rare muscular fibers of irregular in size, with an excess of conjunctive tissue (**Fig EV1I-K**).
