# Supplementary figures and images for: Pathogenic PDE12 variants impair mitochondrial RNA processing causing neonatal mitochondrial disease
Source: EMBO Mol Med. 2024 Nov 20;17(1):193–210. doi: 10.1038/s44321-024-00172-5 (PMC11729904; doi:10.1038/s44321-024-00172-5)

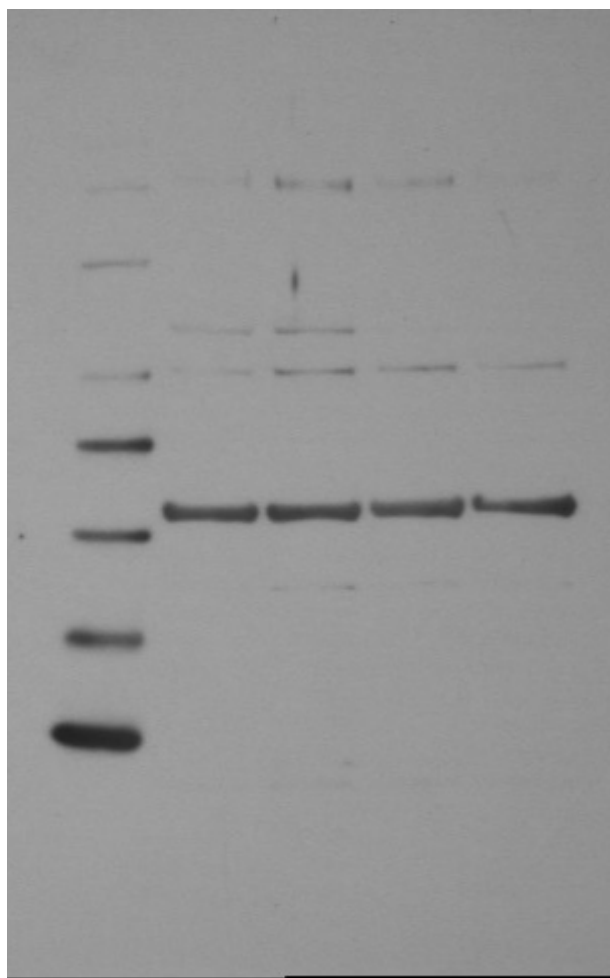

← Actin  
~42kDa

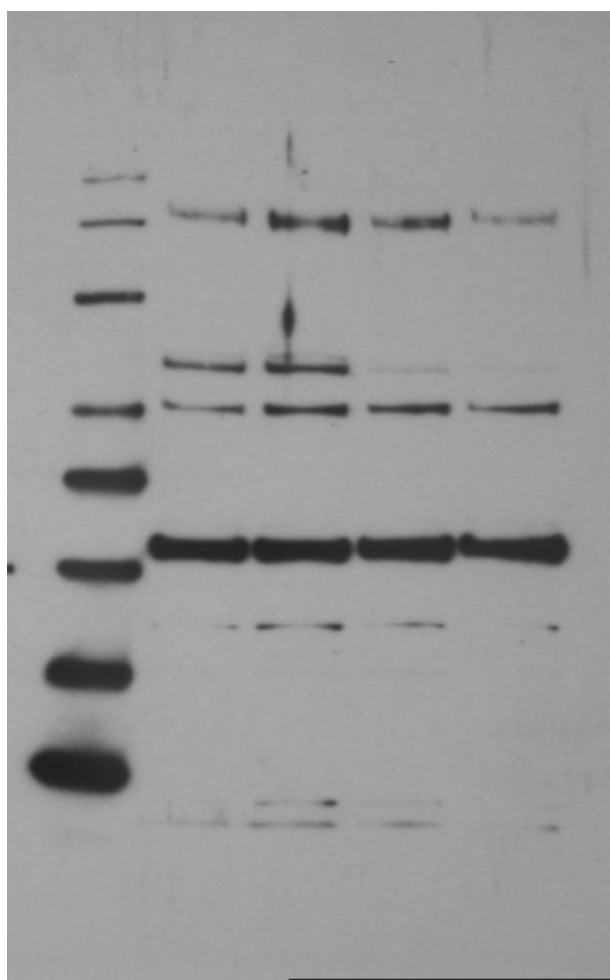

← PDE12  
~65kDa

Supplement: Supplementary file 3 — Source data Fig. 3 [file 44321_2024_172_MOESM3_ESM.zip › EMM-2024-19766_SourceDataForFigure3A.pdf]

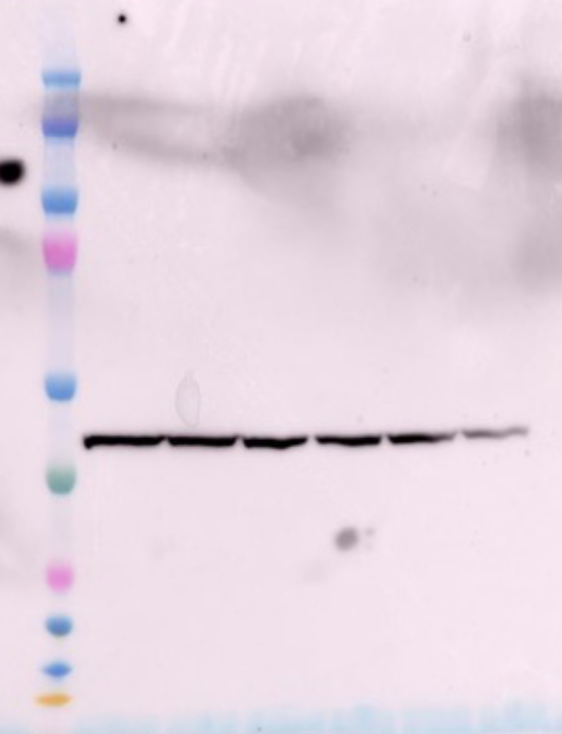

anti-Beta-actin

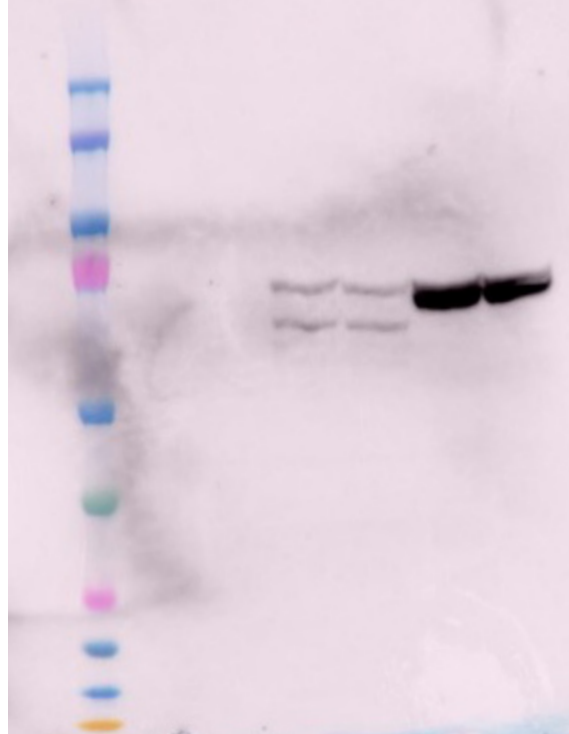

anti-FLAG

Supplement: Supplementary file 3 — Source data Fig. 3 [file 44321_2024_172_MOESM3_ESM.zip › EMM-2024-19766_SourceDataForFigure3C.pdf]

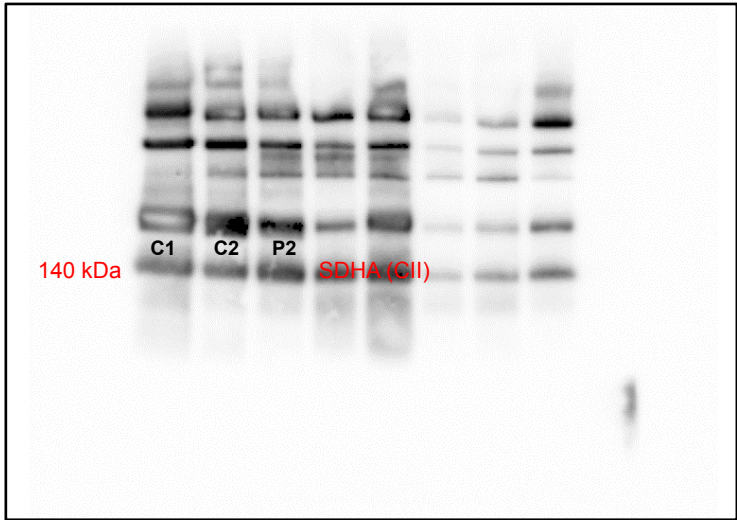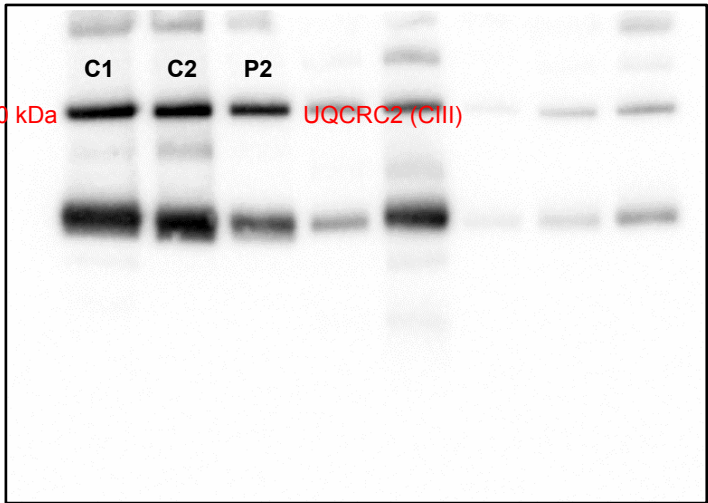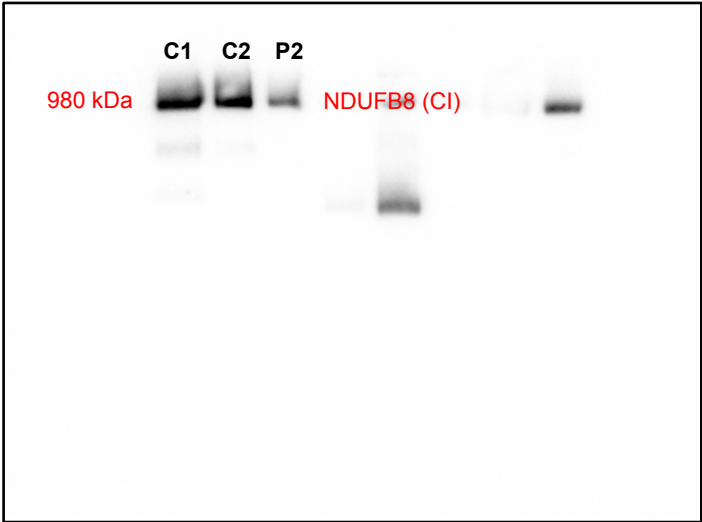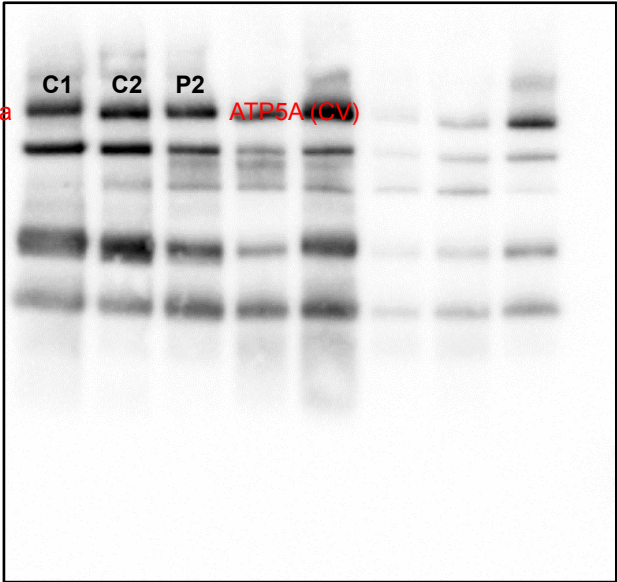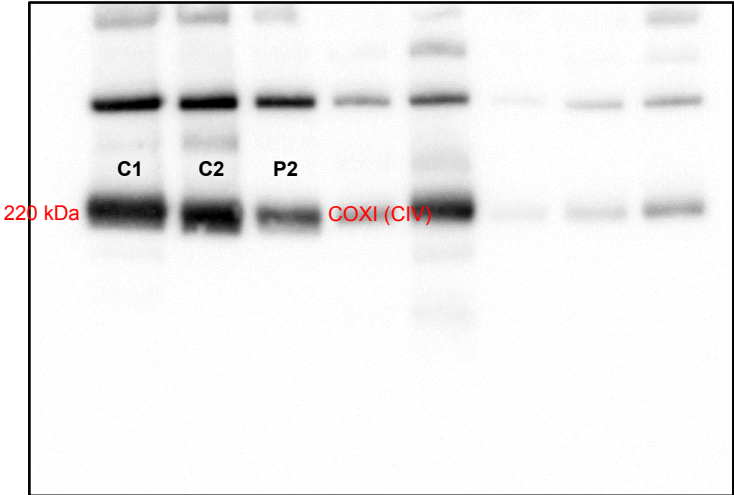

Supplement: Supplementary file 6 — Source data Fig. 6 [file 44321_2024_172_MOESM6_ESM.zip › EMM-2024-19766_SourceDataForFigure6A.pdf]

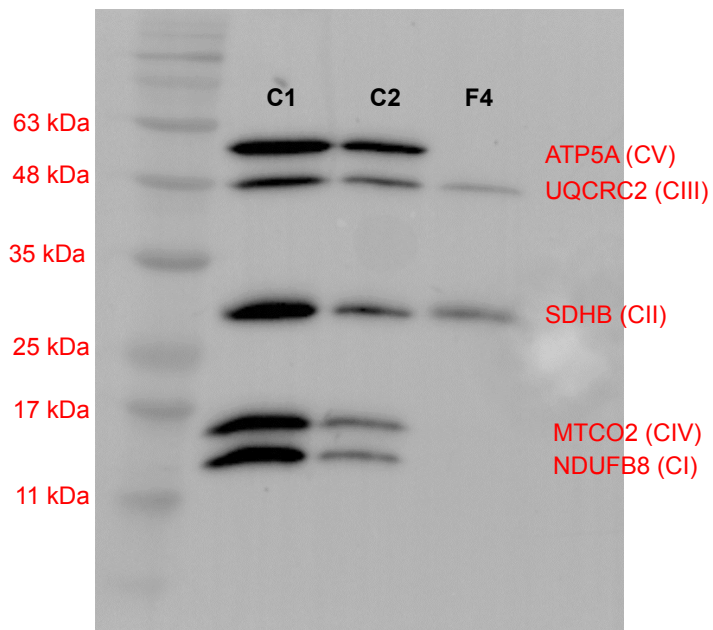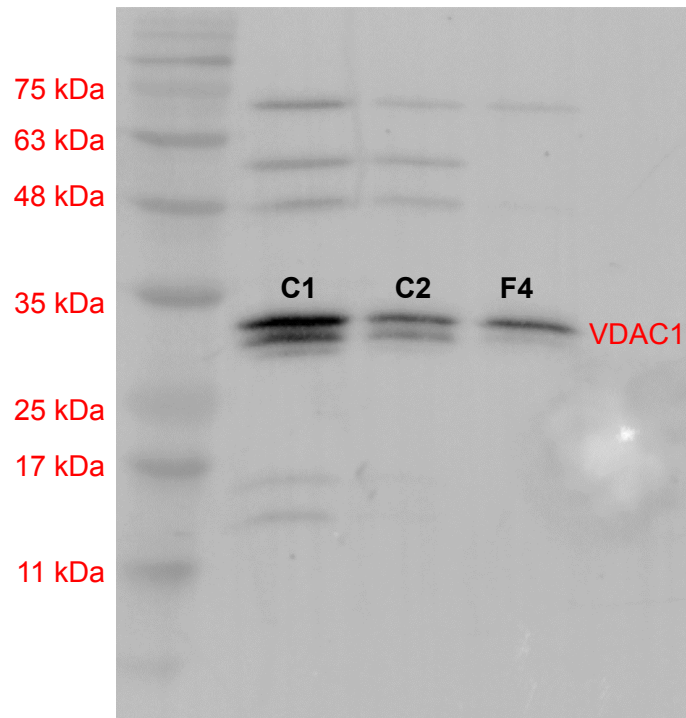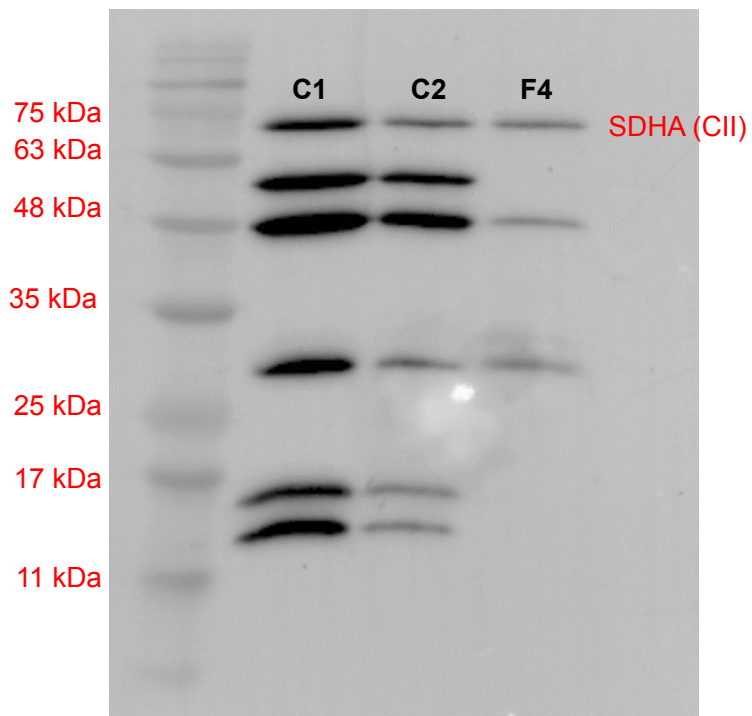

Supplement: Supplementary file 6 — Source data Fig. 6 [file 44321_2024_172_MOESM6_ESM.zip › EMM-2024-19766_SourceDataForFigure6C.pdf]

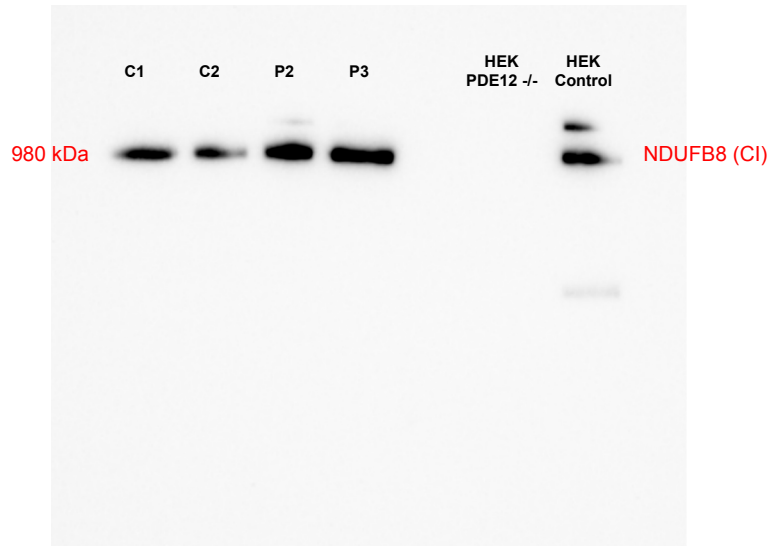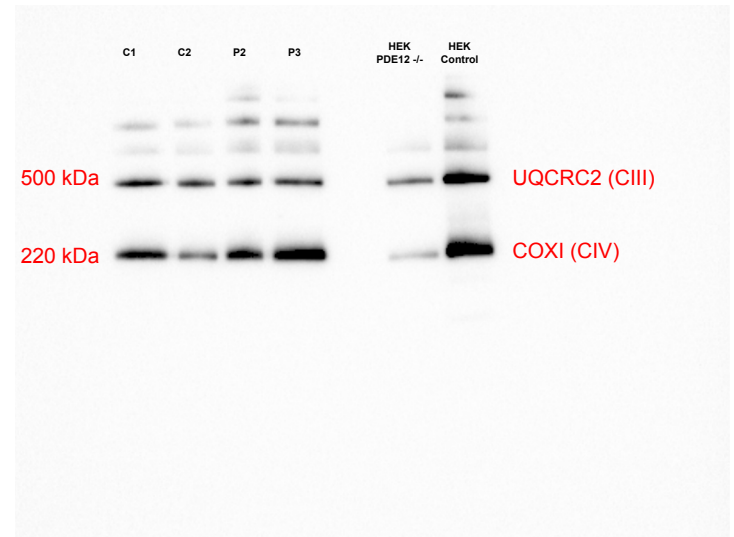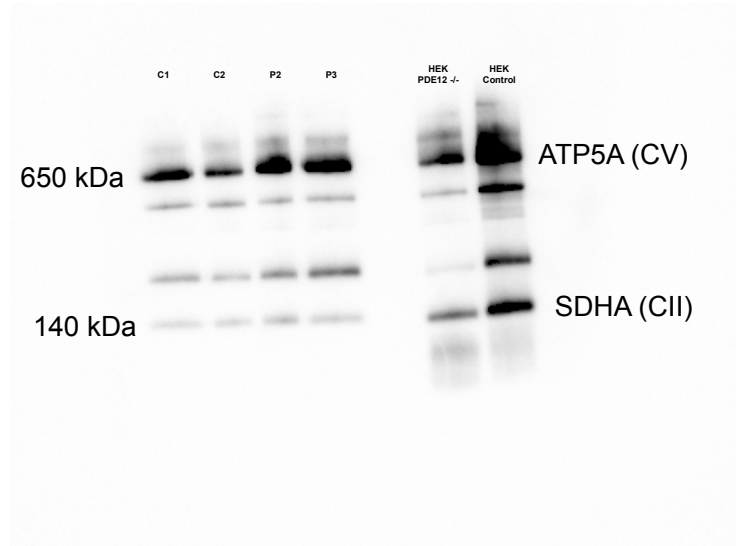

11.1s exposure

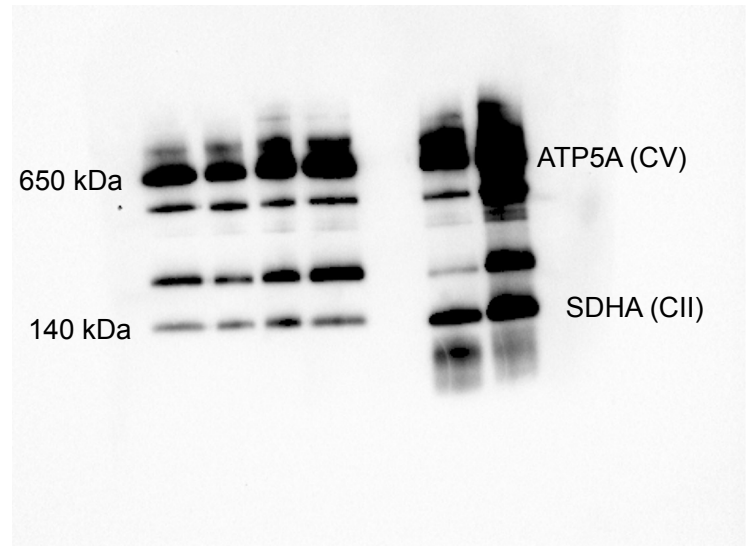

39.4s exposure

Supplement: Supplementary file 6 — Source data Fig. 6 [file 44321_2024_172_MOESM6_ESM.zip › EMM-2024-19766_SourceDataForFigure6E.pdf]
