# Supplementary material for: Pathogenic PDE12 variants impair mitochondrial RNA processing causing neonatal mitochondrial disease
Source: EMBO Mol Med. 2024 Nov 20;17(1):193–210. doi: 10.1038/s44321-024-00172-5 (PMC11729904; doi:10.1038/s44321-024-00172-5)
Supplement: Supplementary file 8 — Expanded View Figures [file 44321_2024_172_MOESM8_ESM.pdf]

Expanded View Figures

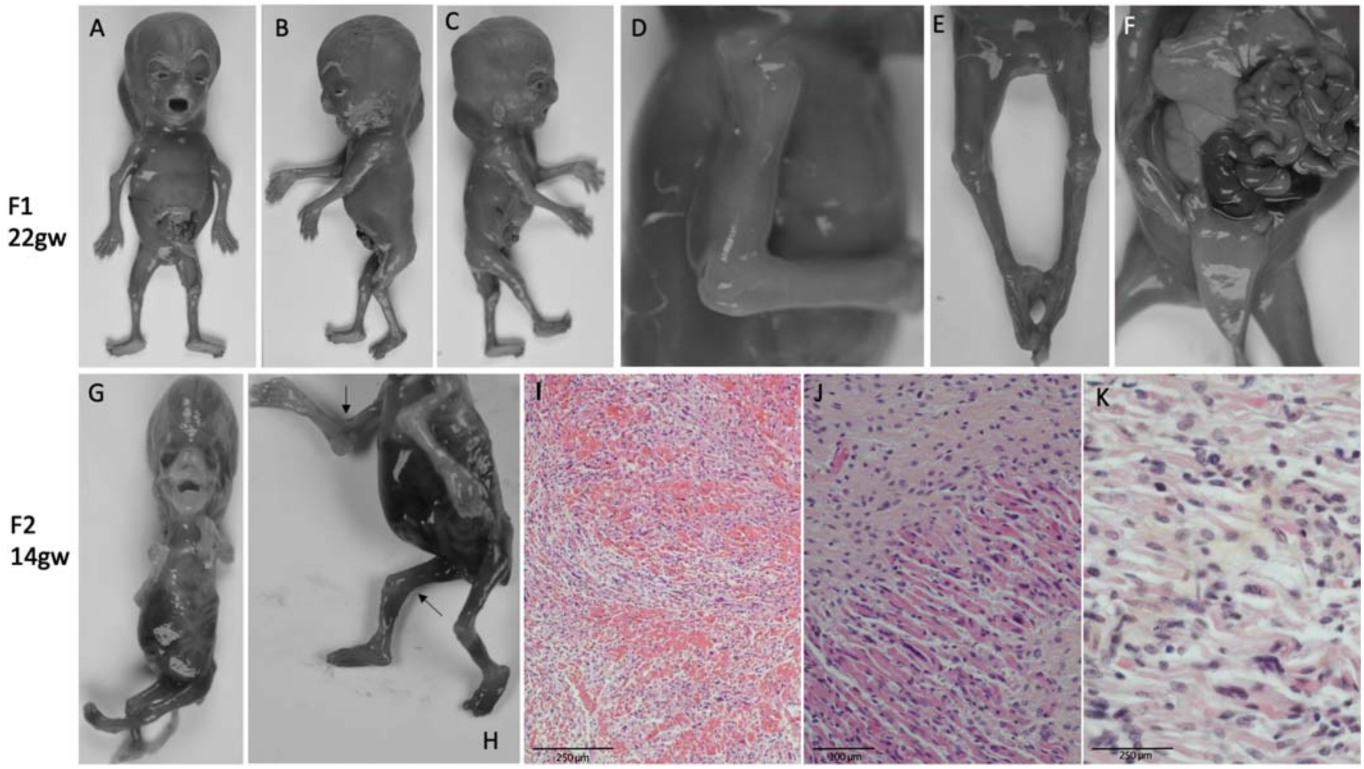

Figure EV1. Images and muscle histology of Foetuses 4 and 5.

Images and muscle histology of Foetus 4 (22 gw, **A–F**) and Foetus 5 (13,2 gw, **G–K**). (**A–C**) Face and profile pictures showing cystic hygroma, facial dysmorphisms with hypertelorism, high and large forehead and low-set ears. (**D**) Right arm showing elbow ankylosis. (**E**) Lower limbs showing bilateral knee ankylosis. (**F**) Autopsy showing pulmonary hypoplasia and distended bladder. (**G**, **H**) Face and profile pictures showing contractures of four limbs with arthrogryposis, multiple pterygia (colli, elbows, knees) and generalised muscular atrophy. (**I–K**) Histological analysis showing rare muscular fibres of irregular in size, with an excess of conjunctive tissue. Both foetuses present intra-uterine growth retardation and hydrops fetalis.

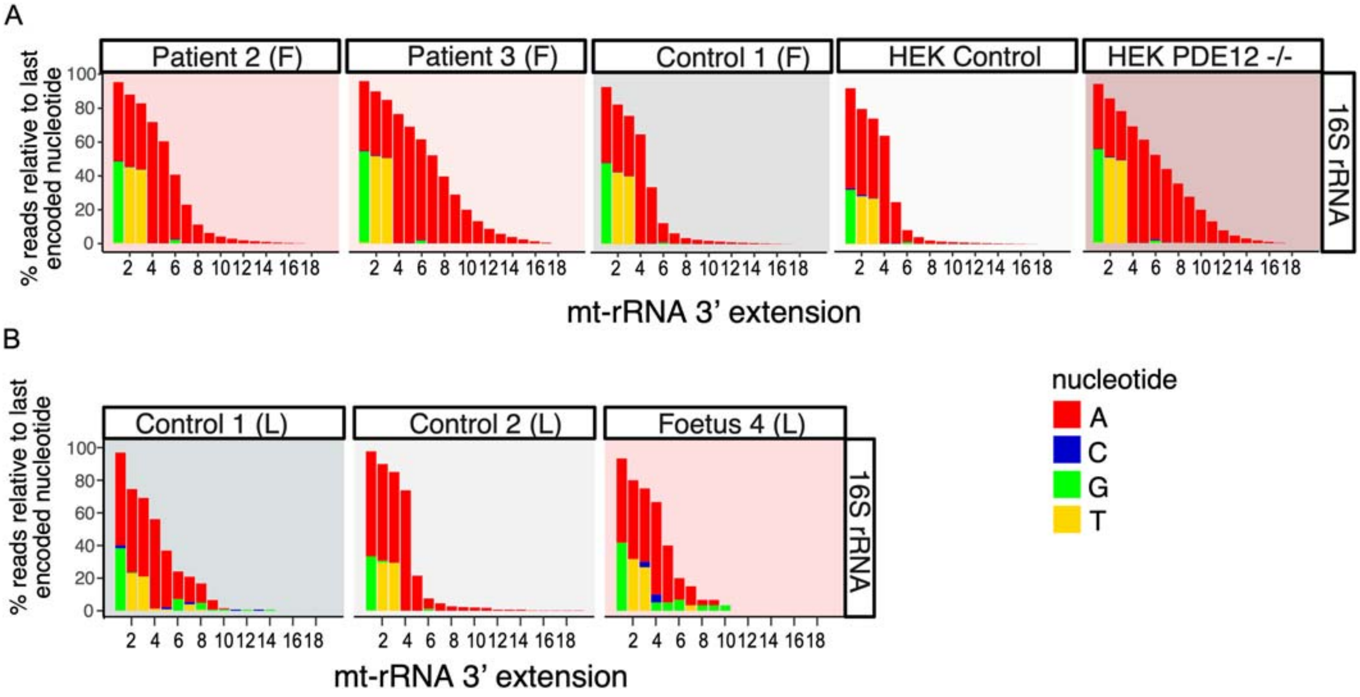

Figure EV2. Spurious 3′ adenylation of 16S rRNA.

(**A**) Representation of reads beyond the encoded nucleotides for 16S rRNA for Patient 2 and Patient 3 fibroblasts, fibroblast control, HEK PDE12 KO cells and control HEK cells ascertained by MPAT-Seq. ($n = 1$). (**B**) Representation of reads beyond the encoded nucleotides for 16S rRNA for RNA extracted from liver of Foetus 4 liver and age-matched control liver ascertained by MPAT-Seq. ($n = 1$). Source data are available online for this figure.

 © The Author(s)

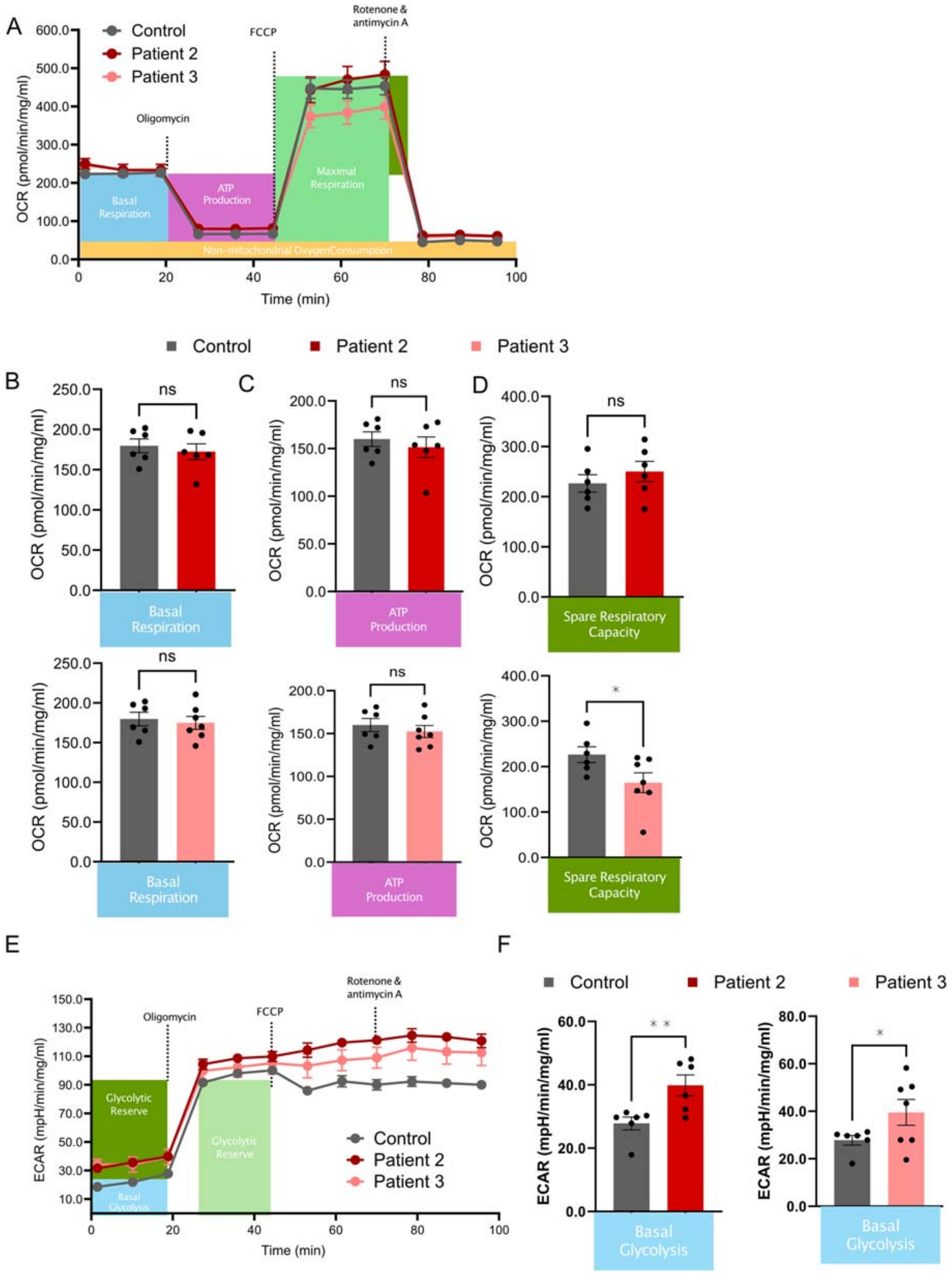

Figure EV3. Mitochondrial respiration in patient fibroblasts.

(**A**) Mitochondrial oxygen consumption rate (OCR) as measured by a flux analyser on control fibroblasts (grey) and fibroblasts of Patient 2 (red) and Patient 3 (pink). (**B**) Basal respiration was measured before the addition of Oligomycin. (**C**) Oligomycin was added to determine ATP production. (**D**) FCCP was added to determine the maximal respiration and spare respiratory capacity (*$p = 0.0263$). (**E**) Extracellular acidification rate. (**F**) Basal glycolysis. Error bars in (**A**–**F**) represent standard error of mean. Each measurement was conducted in at least three replicates (*$p = 0.0427$, **$p = 0.0055$). The one-tail t-test was used to calculate the p values. Dots show individual measurements ($n = 6$, technical replicates).

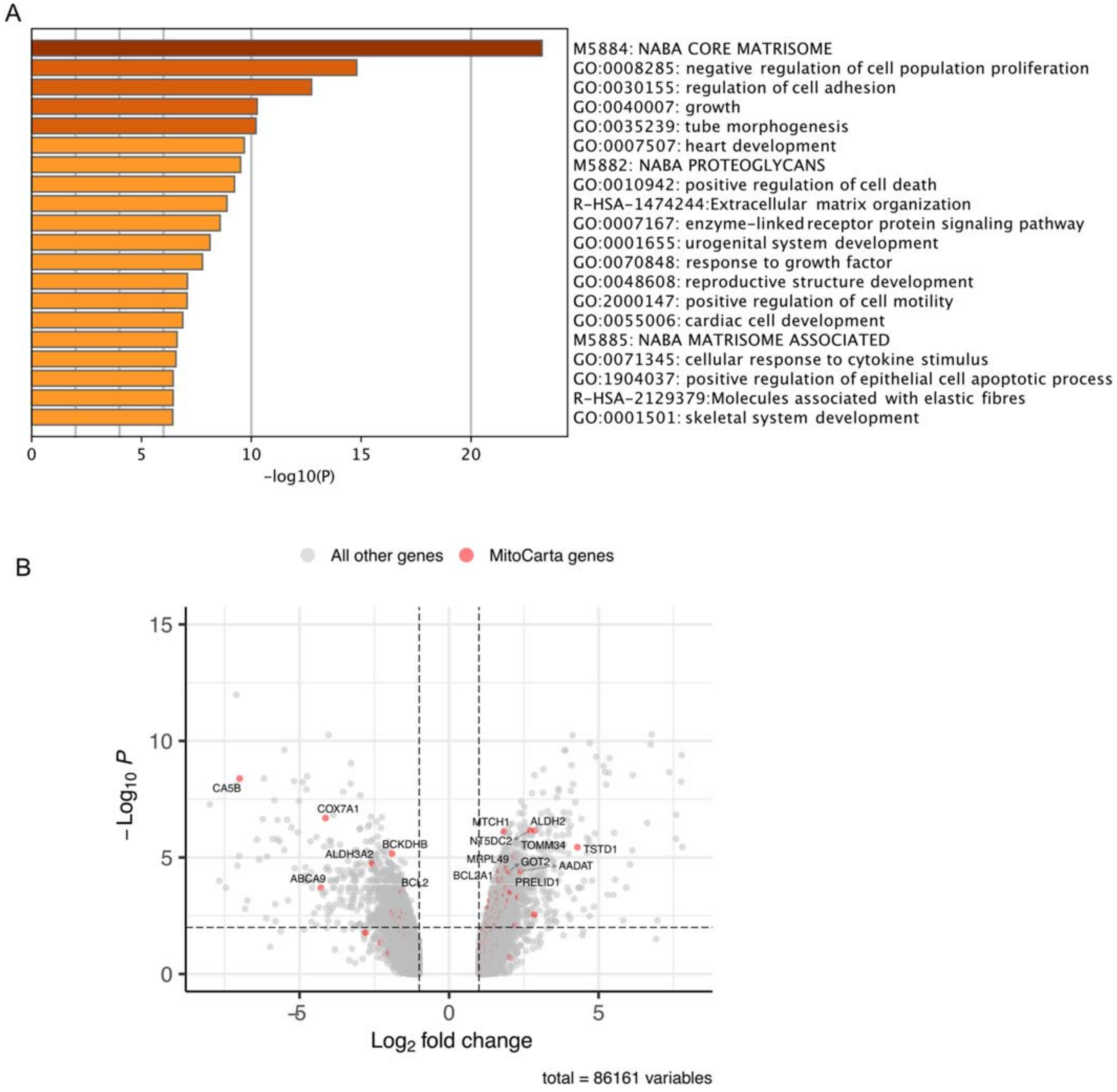

Figure EV4. Gene expression analysis in patient fibroblasts.

(**A**) System-level analysis of Affymetrix Clariom D transcriptome arrays comparing the RNA levels in Patients 2 and 3 with unaffected controls. Statistical analysis was done using the ANOVA test and FDR-corrected values. The multiple-testing correction is based on the approach of Benjamini, Hochberg, and Yekutieli. (**B**) Volcano plot showing the differences in gene expression between RNA from Patients 2 and 3 compared with unaffected controls. Red dots represent genes that localise inside the mitochondria, according to MitoCarta, with the most significantly altered genes labelled. Statistical analysis as described for EV4A. ($n = 1$ biological replicate).

 © The Author(s)
